# Are we comfortable managing oral anticoagulation at the end of life? A national survey of secondary care clinicians in the UK

**DOI:** 10.1016/j.clinme.2025.100505

**Published:** 2025-08-23

**Authors:** Thomas Shevlin, Michelle Kidd, Hannah Cronin, Alastair Gilmore, Catherine Hayle, Elizabeth Jones, Rachel Parry, Rose Penfold, Olga Tsiamita

**Affiliations:** a Mid-Cheshire Hospitals NHS Foundation Trust, Crewe, Cheshire, UK; b Wirral University Teaching Hospital NHS Foundation Trust, Wirral, Birkenhead, UK; c Usher Institute, University of Edinburgh, Edinburgh, UK; d Royal London Hospital, Barts Health NHS Trust, London, UK

**Keywords:** Palliative care, Thrombosis, Haemostasis, Anticoagulation, Medico-legal

## Abstract

**Aims/Background:**

Prior research has demonstrated variation of opinion among physicians regarding management of oral anticoagulation (OAC) at the end of life (EOL). To investigate current practice and attitudes towards OAC at the EOL among secondary care clinicians in the UK, with particular emphasis on how comfortable clinicians feel in making these decisions.

**Methods:**

A digital, self-report survey was designed, validated by experts in relevant fields and then distributed via email to secondary care clinicians in the UK involved in EOL care, utilising the existing mailing lists of national societies and research groups.

**Results:**

186 responses were received across 10 specialties and 47 NHS trusts. 56% of clinicians routinely seek advice from colleagues regarding these decisions. 53% of respondents reported having worried about the medico-legal implications of discontinuing OAC, 52% of whom reported that these worries influence their decision making. Patient characteristics are more commonly considered than anticipated life expectancy in deciding whether to suspend or continue OAC at the EOL (56.5% versus 43.5%), 83% of respondents would feel more confident in broaching this subject if national guidance existed.

**Conclusions:**

Our findings demonstrate a significant variation in practice, a widely reported discomfort and a strong desire for professional guidance regarding the management of OAC at the EOL, among UK-based secondary care clinicians.

## Highlights

• Most clinicians responding to this survey, except for those working in oncology, valued patient characteristics such as swallow ability and treatment side effects over life expectancy when reviewing OAC in the EOL.
• Most respondents to this survey routinely seek advice from colleagues regarding a decision to suspend OAC in patients approaching the EOL, mainly from colleagues in the same team or responsible clinician caring for the patient’s life-limiting diagnosis.
• Over half of clinicians surveyed report having worried about the medico-legal implications of either continuing or discontinuing OAC. This rate was the highest in non-medical professionals and the lowest in consultants. Over half of the above, report that these worries influence their clinical decision-making.
• When directly asked, most clinicians agreed that national guidance would help them feel more confident in broaching this subject, but have highlighted the risk of forfeiting individualised care.

## Graphical abstract

**Figure:**
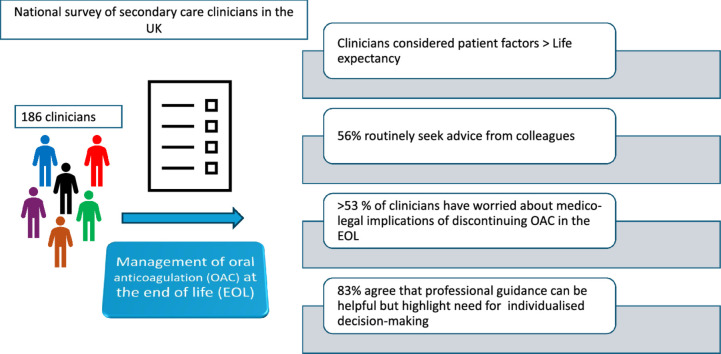

## Introduction

Reforming EOL care has been made an NHS priority, with the British Geriatrics Society highlighting the need for holistic, multidisciplinary care with effective health communication pathways and a focus on de-medicalisation of death.^1^ NHS England and the National Palliative and End of Life Care Partnership have published a national framework that sets six key ambitions for care, two of which refer to prioritising individualised care and regular reviews to minimise distress.^2^ Oral anticoagulants (OAC) are considered high-risk medicines by the Care Quality Commission, in view of their associated risk of bleeding – a risk that only becomes acceptable when counterbalanced by a higher risk of arterial or venous thrombosis.^3^ OACs include both vitamin K antagonists (VKA) and direct oral anticoagulants (DOAC), with the latter found to have a more favourable bleeding risk profile in the frail population while maintaining efficacy in venous thromboembolism (VTE) prevention.^4^ The specific OAC used, its dose and desired therapeutic range, as well as patient comorbidities, concomitant medication and individual patient characteristics (such as weight, frailty and lifestyle) are all determinants of overall bleeding risk and, accordingly, many of these factors are captured by validated bleeding risk prediction tools, including the HAS-BLED score.^5^

In one study of older patients (>65 years old) in Sweden, antithrombotics, encompassing both antiplatelets and OAC, were found to be the second most prescribed class of drugs in the final month of life,^6^ a finding at least partially explained by the higher prevalence of conditions such as atrial fibrillation and VTE in this age group. Nevertheless, the decision whether to continue or suspend anticoagulation in patients with limited life expectancy is a nuanced one, requiring an individualised approach that involves a risk–benefit assessment and due consideration for the patient’s perspective, lifestyle and priorities for quality of life. Some of the challenges identified in physician and patient surveys on OAC patients with a limited life expectancy are the lack of evidence on benefit versus harm of discontinuing anticoagulation in this context, uncertainty about the patient’s life expectancy, burden of the decision^7^ and a wide variation in decision-making perceptions among patients and physicians alike.^8^

To tackle some of the challenges of de-prescribing, the Royal College of Physicians has published a toolkit that highlights the risk of harmful polypharmacy in any patient receiving EOL care who takes four or more medications and flat anticoagulants as especially ‘problematic’, given their narrow therapeutic index.^9^ Several other decision-support tools for deprescribing, with particular focus on risk patient groups such as the frail and older population, have been developed by NHS England and NHS Specialist Pharmacy Service,^10^ albeit not directly referring to anticoagulation review at the EOL. One de-prescribing scheme specifically aimed at antithrombotics in patients with solid cancers approaching the EOL has been piloted in a single-centre study in the Netherlands and deemed safe, with no increase in thrombotic events observed in the de-prescribing scheme group.^11^

Malignant disease, wherein an increased risk of both venous thromboembolic disease and anticoagulation-associated bleeding exist inextricably, poses a unique clinical challenge.^12^ Furthermore, existing literature, albeit limited in size and quality of evidence, suggests that, towards the EOL, anticoagulation confers a significant risk of bleeding without necessarily reducing the risk of thromboembolic events.^13^

To some, the symptomatic burden of acute thromboembolism, and the prolonged and symptomatic death it confers, affords anticoagulation a role in palliation.^8^ However, the significant risk and burden of anticoagulation-associated bleeding in this pharmacokinetically fragile cohort cannot be understated.^14^

## Aim

To gauge current practice and attitudes towards oral anticoagulation (OAC) decisions at the end of life (EOL) among secondary care clinicians in the UK, with particular emphasis on factors influencing decision-making, clinician’s perceptions and differences in practice between specialty and grade of training.

## Material and methods

A pilot survey (Supplementary Appendix, S1) was distributed to eight consultants across haematology, palliative medicine, geriatrics and acute internal medicine, who were asked to rate the validity, clarity and relevance of each question stem. This survey was subsequently modified when content validity, clarity and relevance ratios failed to meet the required threshold for the sample size, as per the Lawshe Table (Supplementary Appendix, S2). Questions and answers were informed by prior research and refined by the expert opinion of this multidisciplinary pilot cohort.^11^^,^^13^

Following validation, a digital survey was designed on Microsoft Forms® (Supplementary Appendix, S3) and distributed via email to secondary care clinicians in the UK involved in EOL care, utilising professional networks of national societies and research groups (Supplementary Appendix, S4). The professional networks utilised were selected as it was postulated that they form a representative sample of clinicians with various levels of experience across secondary care specialties, which include caring for patients with limited life expectancy with frequently encountered dilemmas pertinent to OAC decisions. Answers were collected between November 2024 and February 2025. The survey did not require formal review by an NHS research ethics committee, according to the Health Research Authority decision tool.^15^

Data were analysed and visualised using the GraphPad Prism version 10.4.2 software.

## Results

### Respondents’ characteristics

186 responses were received from secondary care medical prescribers across all grades (40% consultants, 37% ST3+ doctors, 10% pre-ST3 doctors) and non-medical clinicians (13%), such as pharmacists and advance care practitioners. All respondents were involved in the management of patients at the EOL across 47 NHS trusts and all devolved nations of the UK over 8 weeks (25 November 2024 to 20 January 2025). Their self-reported field of practice was in haematology (30%), geriatric medicine (18%), general medicine (12%), oncology (12%), palliative medicine (12%), acute medicine (10%) and other including emergency care (6%).

### Clinicians’ practices

**Fig. 1:**
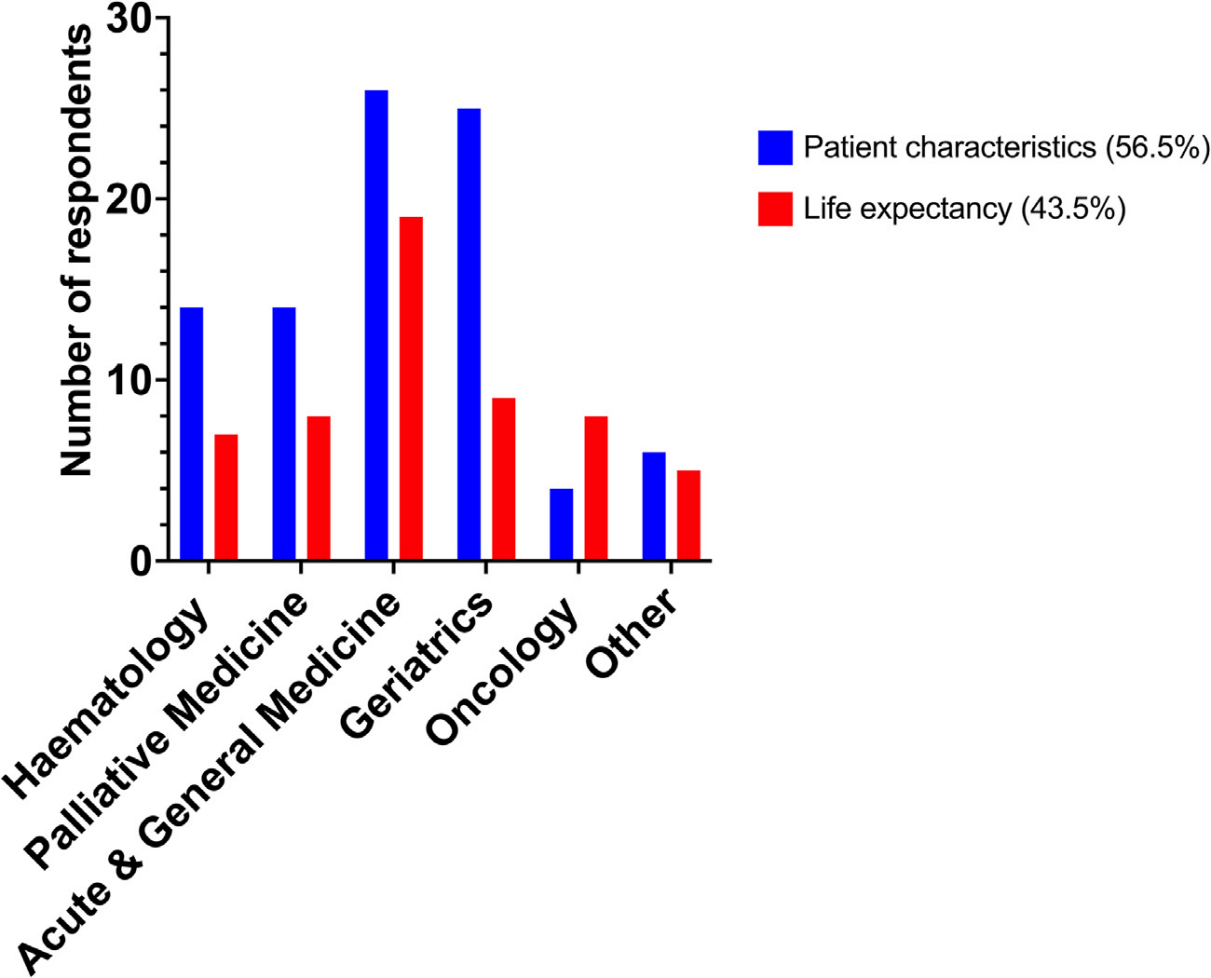
Distribution of main factors considered by clinicians according to specialty.

Clinicians were asked whether they consider patient characteristics, such as swallowing ability and treatment side effects, or anticipated life expectancy to be more important when reviewing OAC at the EOL. Most of the respondents (56.5%) valued patient characteristics over life expectancy (43.5%) when making this decision and were equally distributed across consultant and non-consultant grades. Of note, this difference became starker in specialties like geriatrics (25 out of 34 respondents), haematology (14 out of 21 respondents), acute and general medicine (26 out of 45 respondents) and palliative medicine (14 out of 22 respondents), while oncology was the only specialty in which life expectancy was considered more frequently than patient characteristics (eight out of 12 respondents) (Fig. 1).

**Fig. 2:**
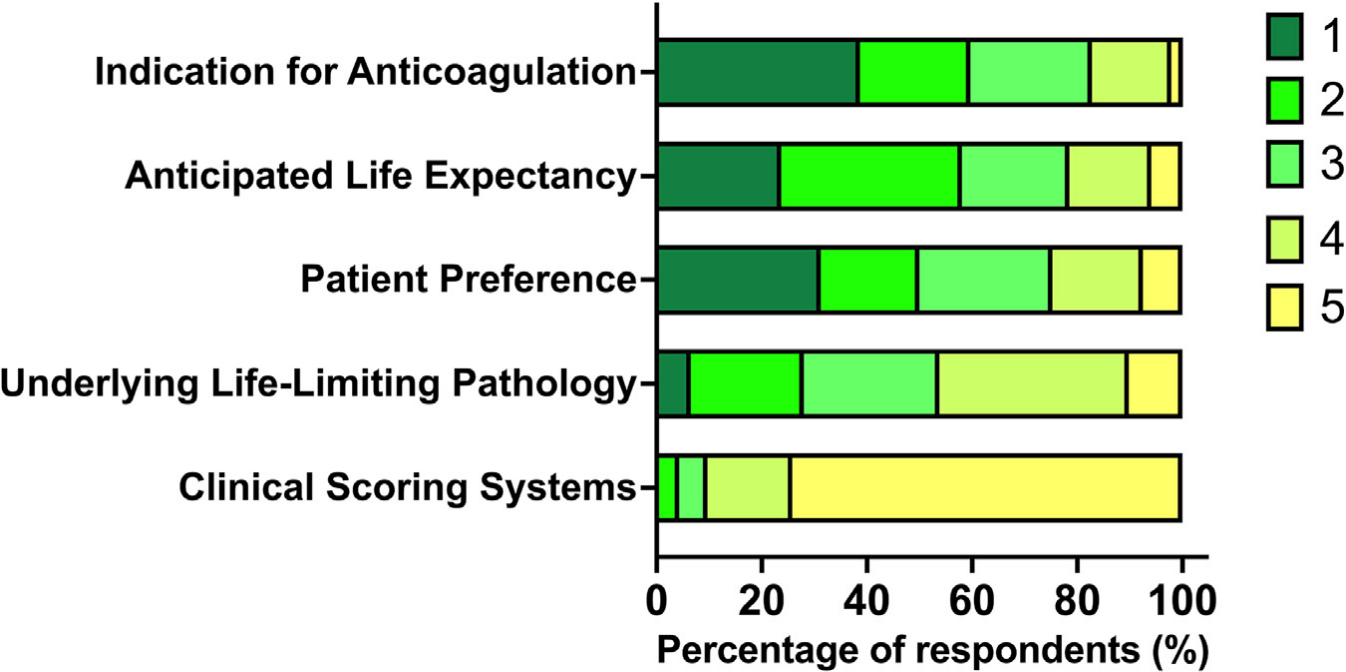
Factors considered by clinicians when deciding whether to discontinue OAC at the EOL, ranked from most (1) to least (5) important.

When asked to rank factors from most to least important in deciding whether to discontinue OAC at the EOL, they ranked clinical indication as the leading consideration, followed by anticipated life expectancy and patient preference. The underlying pathology leading to the life-limiting diagnosis and clinical scoring systems (eg CHADSVASc and HASBLED) were considered the least important parameters (Fig. 2), ranked as fourth or fifth (least important) by the majority of the respondents.

It is worth noting that 48% of clinicians in general medicine, 44% in acute medicine and 38% in haematology considered patient preference to be the most important factor in making these decisions.

Respondents reported being more likely to suspend OAC that requires blood monitoring (eg warfarin) than one that does not (63% versus 35% for equally likely) and felt more comfortable suspending OAC for the prevention of thromboembolism in atrial fibrillation (AF) than in patients with a history of recurrent thromboembolism. Primary prevention of thromboembolism in mechanical heart valves, followed by treatment of acute thromboembolism, were the indications with which clinicians felt least comfortable suspending OAC.

When faced with a clinical scenario of a 75-year-old inpatient taking edoxaban for AF (CHA_2_DS2-VASc score of 4) with an anticipated life expectancy of a few weeks, most clinicians (139/186) would suspend the previously prescribed non-VKA OAC, while a minority (4/186) would permanently discontinue it. The remaining responders opted to either switch to prophylactic (18/186) or treatment dose (9/186) of low-molecular-weight heparin or were unable to decide based on the information provided.

### Clinicians’ attitudes

Overall, 44% of clinicians do not routinely seek advice from another colleague when reviewing OAC at the EOL. In all other cases, it is the clinical team caring for the patient’s underlying life-limiting diagnosis or a colleague in the same team who were the most reported sources of advice. Palliative care physicians were reported to be contacted for support with this matter three times as often as haematologists.

Among doctors, there was an inverse relationship between seniority and the likelihood of seeking advice from a colleague: 30% of consultants, compared to 61% of ST3+ doctors seek advice.

**Fig. 3:**
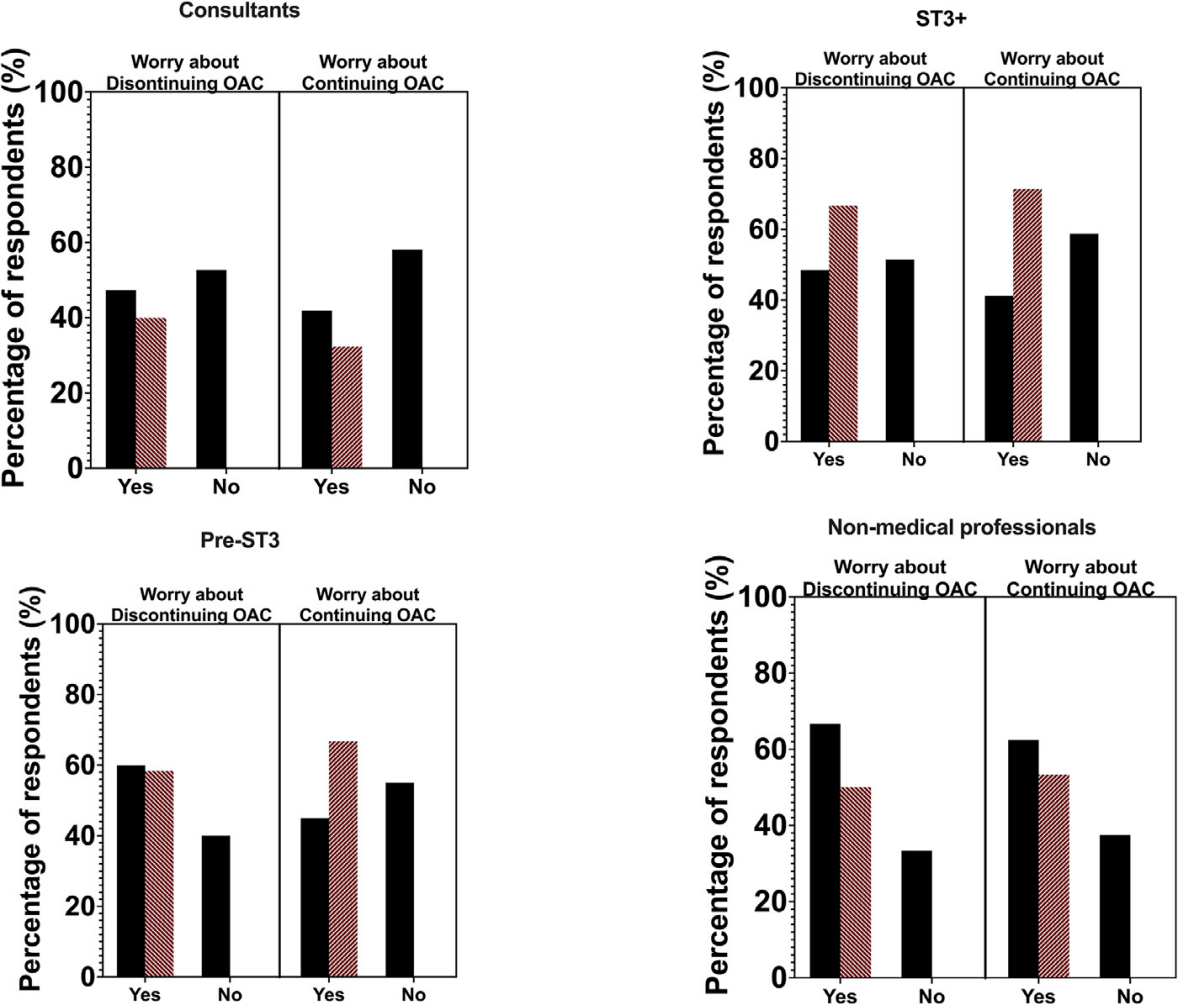
Proportions of healthcare professionals per grade and likelihood of concerns about medico-legal implications of continuing and discontinuing OAC at the EOL (black bars). The red bar expresses the percentage (%) of healthcare professionals worried who feel this concern influences clinical decision-making.

Overall, 52% of respondents reported having worried about the medico-legal implications of discontinuing OAC in the end of life, 53% of whom reported that these worries influence their decision making. Worry was not limited to discontinuation of OACs, however, with 44% of respondents reporting that they worry about continuing OAC in this setting, among whom 53% reported that these worries affect their decision making. OAC discontinuation concerns were more likely to influence decision making in non-medical professionals (63%), pre-ST3 doctors (60%) than ST3+ doctors (49%) and consultants (47%), with a similar pattern noted when deciding to continue OAC (Fig. 3).

**Fig. 4:**
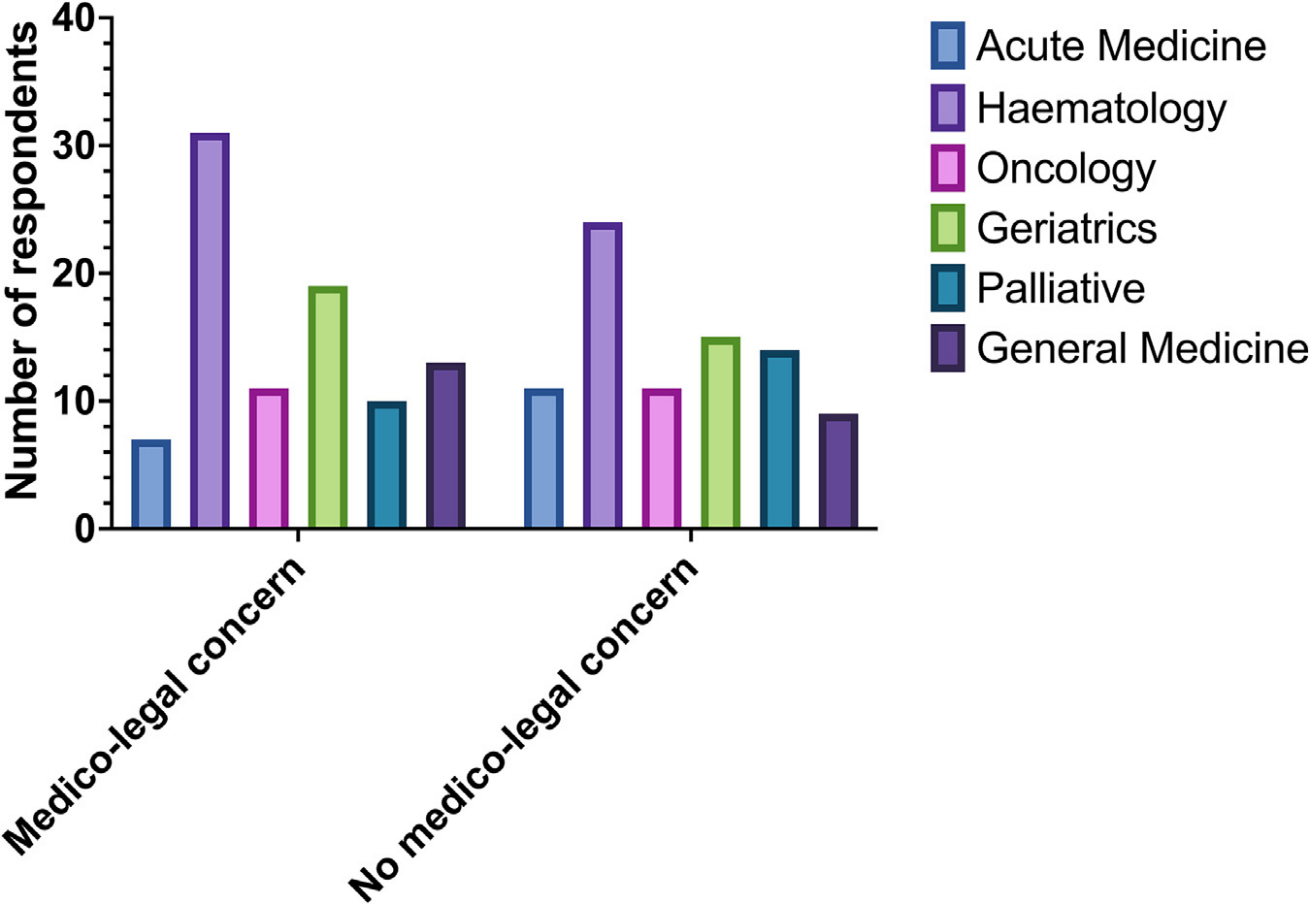
Number of respondents per specialty feeling concern for medico-legal implications of discontinuing anticoagulation at the EOL.

ACP/ANPs and pharmacists were more likely to worry about the medico-legal implications of discontinuing and/or continuing OAC than doctors of any grade. When divided by specialty group, haematologists were most likely to experience medico-legal concerns when discontinuing anticoagulation at the EOL (31 out 55 of respondents working in haematology), while those working in palliative care were the least likely to report these concerns (10 out of 24 respondents in palliative medicine). Fig. 4 illustrates the relevant frequencies for each specialty group.

When asked whether they would feel more confident in broaching discussions about OAC at the EOL if national guidance on this existed, 83% of respondents agreed. Free-text comments from some clinicians mostly included concerns that a consensus cannot replace the highly individualised, holistic decisions that should be taken jointly with patients. One respondent mentioned that the evidence base is not sufficient and the diversity of EOL circumstances is too vast to be accounted for in such a guidance. A list of all the qualitative feedback on an OAC at the EOL guidance can be found in the Supplementary Appendix (S5).

## Discussion

To our knowledge, this is the first UK national survey to include responses from both medical and non-medical prescribers, as well as doctors of different grades, to explore their attitudes and confidence regarding the use of OAC in patients approaching the EOL irrespective of their life-limiting diagnosis. It is worth noting that clinicians can, of course, make and communicate clinical decisions confidently, even while experiencing discomfort with those decisions, owing to the patient’s cultural influences at play, and their impact on interaction between clinicians and patients, at the EOL.^16^

Though in many cases, patients on OAC are recognised as terminally ill or spend their last few days of life in hospital,^17^ recognising the first signs of the dying process and choosing an appropriate time to review and rationalise medication and care goals is subjective and, sometimes, challenging. The GMC’s Treatment and Care Towards the EOL guideline defines patients as ‘approaching the EOL’ when they are likely to die within the next 12 months. Our findings suggest that finely tuned decisions on whether to discontinue OAC can be taken by a variety of clinicians with a wide range of training experience and specialisations and elucidates the subjective variability involved in the decision-making process. Indeed, existing evidence suggests that clinicians working in primary care compared to cardiologists working in secondary care make different decisions on antithrombotics and are less likely to prescribe them for atrial fibrillation.^18^ Furthermore, preferences and considerations may differ significantly between patients and healthcare staff, a possibility corroborated by our finding of variability in the decisions made by different clinicians when faced with the same hypothetical clinical scenario. Moreover, while in this survey, clinicians were more likely to discontinue OAC requiring blood monitoring such as warfarin, evidence from a qualitative study in 2015 suggests that, from the patient’s perspective, this is a minor issue that may not influence their choice of anticoagulation.^19^ Overall though, the lack of requirement of blood monitoring with DOACs, in combination with their favourable safety profile, must surely offer a practical advantage compared to VKA for frailer patients requiring therapeutic anticoagulation.

The most commonly reported reasons for OAC discontinuation in this setting given by respondents of this survey – namely, development of bleeding complications or unsafe swallow – corroborate those of a Dutch chart study in which 76% of patients continued antithrombotics until their last week of life.^17^ Interestingly, one recent cohort study found that patients entering their last year of life experienced similar rates of thromboembolism and bleeding events, regardless of whether the VKA was continued or not,^20^ while another found that the quality of anticoagulation decreased due to higher times above the desired therapeutic range, particularly in the last 3 months of life.^21^ Despite the lack of direct comparison of DOACs to the VKA in this setting, similar results of uncertain benefit at the EOL when continuing any type of anticoagulation were observed in a cohort study, where patients who discontinued anticoagulation were more likely to have a home death as opposed to dying in a healthcare facility.^22^

Despite having clear indications and guidelines for when to commence anticoagulation, there is no such guidance for discontinuing it. Our findings confirmed that secondary care prescribers often seek advice from other colleagues, most frequently from those in the same clinical team or the coordinating team caring for the patient’s life-limiting diagnosis. When consulting another specialty, palliative medicine physicians were the most commonly reported source of advice. As expected, this was less often reported by consultants than early-career residents and non-medical prescribers. That said, a recent study in Canada showed that it is palliative medicine specialisation rather than time from medical qualification that is associated with higher rates of anticoagulation discontinuation at the EOL.^22^ Indeed, the need to support with and consider deprescribing including antithrombotic medication in all patients receiving palliative care is long recognised, with an antithrombotic deprescribing tool already developed for patients seen by palliative team professionals with terminal cancer in the Netherlands.^11^ Most importantly, the integral role of patient and public involvement in this field has been exemplified and put into practice by an ongoing multinational research project with the aim of developing a shared decision tool to support cancer patients, their loved ones and healthcare staff with antithrombotic management at the EOL.^23^^,^^24^

Interestingly, worry about medico-legal implications was more commonly reported with respect to discontinuing OAC than continuing it. Considering the tendency towards bleeding without reduction in thromboembolic risk in this patient cohort,^12^^,^^25^ as well as a propensity for coroners to find use of anticoagulation to contribute to more deaths than disuse,^26^ it would appear that this concern is misplaced. Findings from a Danish cohort study indicate that approximately 12.5% of patients are on an OAC when a terminal diagnosis is made and majority of those continue this until death.^27^ In the same study, patients who remained on antithrombotics until the EOL experienced lower VTE risk, albeit at the expense of a higher risk of major bleeding. Specialties such as acute medicine and oncology had lower reported rates of these concerns, perhaps due to having more experience in decision making in a higher-risk, acute setting or having less experience of caring for frail patients with multiple chronic comorbidities. Of note, haematologists, despite their expertise in bleeding and thrombotic disorders, experienced higher rates of concern on the medico-legal implications of their decisions, a finding possibly explained by their involvement in more complex cases or in the investigation of incidents when patients have suffered harms of over- or under-anticoagulation.

Clinical guidance can be a double-edged sword, and care must be exercised to avoid publication of advice that is overly restrictive. Feedback from the survey respondents indicates that while most colleagues (83%) would find some consensus guidance useful, others reported that drafting guidance for this scope of practice could be impossible or even harmful. Nevertheless, guidance such as in the form of a decision-support tool could provide a framework for shared decision-making and a structured OAC review at the EOL by highlighting indications for discontinuation and signposting patients and clinicians to existing published resources and required bedside assessments.^28^ For example, concepts such as time to benefit become much relevant in a patient with limited life expectancy and an awareness of the time to benefit for each OAC indication could be key to deciding when to review OAC and help illustrating the rationale for this clinical decision to patients.^29^

The main limitations of this survey are its relatively small sample size and our inability to calculate non-response bias as the survey was widely distributed via multiple channels – namely both national professional networks and hospital physicians’ local specialty teams – which, alongside the lack of sample size calculation, prevents the generalisation of the survey findings. Furthermore, the validation cohort only included consultant-grade physicians in view of their expert feedback, but no representation of other healthcare professionals which were included in the main survey population sample. Of note, a recent European flash-mob survey on anticoagulation deprescribing patterns had a similar number of respondents and yielded results consistent with our findings of variation in practice in this setting.^30^ What’s more, the survey discussed indications for anticoagulation in broad terms, without using specific thrombotic risk scoring systems or individualised scenarios, allowing room for subjective interpretation by the respondents. Since most questions did not allow a free-text option, the survey was unable to elucidate the nuance behind decision making in this area and the closed questioning used throughout could be considered leading and give the illusion that medico-legal concern is the only factor behind clinicians’ worries when making high-risk decisions, which is unlikely.

Finally, we acknowledge that secondary care physicians are not the main stakeholders in this field of practice, where patients and primary care physicians have a central role in advance care planning and all relevant decision-making dilemmas and should be included in any efforts to develop decision-support tools, guidance or educational resources in this field.

## Conclusions

Our findings suggest a variation in practice, with over half of the respondents agreeing that they have experienced medico-legal concerns when reviewing OAC at the EOL, more so for discontinuing than continuing OAC; a concern that is likely misplaced. While most respondents agreed that national guidance may support decision-making in this field of practice, they highlighted the need for EOL treatment decisions to remain highly individualised. How to best support clinicians to make timely, well-informed and not simply algorithmic decisions regarding OAC at the EOL remains a subject in need of more research.

## CRediT authorship contribution statement

**Thomas Shevlin:** Writing – review & editing, Writing – original draft, Visualization, Methodology, Data curation, Conceptualization. **Michelle Kidd:** Writing – review & editing, Validation, Methodology. **Hannah Cronin:** Writing – review & editing, Validation, Methodology. **Alastair Gilmore:** Writing – review & editing, Validation, Methodology. **Catherine Hayle:** Writing – review & editing, Validation, Methodology. **Elizabeth Jones:** Writing – review & editing, Validation, Methodology. **Rachel Parry:** Writing – review & editing, Validation, Methodology. **Rose Penfold:** Writing – review & editing, Validation, Methodology. **Olga Tsiamita:** Writing – review & editing, Writing – original draft, Visualization, Methodology, Data curation, Conceptualization.

## Declaration of competing interest

The authors declare that they have no known competing financial interests or personal relationships that could have appeared to influence the work reported in this paper.

## Appendix Supplementary materials

Image, application 1

Image, application 2
