# Supplementary material for: Are we comfortable managing oral anticoagulation at the end of life? A national survey of secondary care clinicians in the UK
Source: Clin Med (Lond). 2025 Aug 23;25(5):100505. doi: 10.1016/j.clinme.2025.100505 (PMC12447989; doi:10.1016/j.clinme.2025.100505)
Supplement: Supplementary file 1 [file mmc1.docx]

**Anticoagulation at the End of Life: Survey (Pilot)**

Thank you for participating in this short survey.

The aim of this survey is to gather data from secondary care clinicians in the UK on their management of anticoagulation in patients at the end of life. We expect the survey to take no more than 5-10 minutes to complete.

We intend to publish the findings of this survey, but your responses will remain entirely anonymous. Your data will be used for research purposes only and you will not be contacted following completion of the survey. You are free to withdraw your responses at any time prior to publication.

1. Are you a secondary care clinician in the UK responsible for making decisions about patients and their medications as they approach the end of their life?
   - Yes
   - No *– end of survey*
2. What is your role?
   - Consultant
   - Registrar
   - Specialty doctor or associate specialist
   - Pre-higher specialty training doctor
   - Advanced nurse or clinical practitioner
   - Other
3. What is your specialty?
4. Where do you work?
5. Is anticoagulation something that you routinely review in patients approaching the end of their life?
   - Yes
   - No
   - It depends (please elaborate)
6. At what point do you consider reviewing anticoagulation in patients approaching the end of their life?
   - Last days of life
   - Last weeks of life
   - Last months of life
   - When the patient can no longer safely swallow
   - When the patient develops a contraindication to anticoagulation (thrombocytopaenia, renal impairment, low body weight, acute bleeding, drug interaction etc.)
   - When reviewing other medications
   - Other (please elaborate)
7. Do you seek advice from colleagues prior to making decisions about anticoagulation in patients approaching the end of their life? If so, who are you most likely to seek advice from?
   - I don’t seek advice from colleagues
   - Clinician responsible for *originally* initiating their anticoagulant (e.g. Stroke Medicine, Cardiology, Haematology)
   - Clinician coordinating care for their life-limiting diagnosis (e.g. oncology or haematology if malignant disease, respiratory medicine if end-stage COPD, cardiology if end-stage heart failure, neurology if end-stage Parkinson’s etc.)
   - Haematologist (even if they have never been involved in the patient’s care)
   - Palliative care
   - Other
8. Please rank, from most to least important, the following factors that you might consider when deciding whether anticoagulation should continue or not at the end of life.
   - Indication for anticoagulation (e.g. AF, mechanical valves, inherited clotting disorder, acute VTE, recurrent VTE, prophylaxis of VTE)
   - Underlying life-limiting pathology (e.g. malignant vs non-malignant disease, decompensated liver disease, CNS or intra-luminal tumours)
   - Clinical scoring systems (e.g. CHADSVASc, ORBIT and HAS-BLED etc.)
   - Anticipated life expectancy
   - Tablet burden from other medications
   - Patient preference
   - Other (please elaborate)
9. Do you worry about the medico-legal implications of discontinuing anticoagulation (e.g. legal action pursued following a potentially preventable stroke or VTE event)?
   - Yes
   - No
10. Do you worry about the medico-legal implications of continuing anticoagulation (e.g. legal action pursued following a potentially preventable or anticipated bleeding event)?
    - Yes
    - No
11. If yes, do these worries about the medico-legal implications influence your decision-making regarding anticoagulation in patients approaching the end of life?
    - Yes
    - No
12. Would you feel more confident in broaching this subject with patients if there was a national or international consensus on the issue (e.g. in the form of a clinical guideline)?
    - Yes
    - No
    - It depends (please elaborate)
13. Alison is a 75-year-old female with a past medical history of atrial fibrillation and invasive ductal carcinoma of the breast with metastases to the liver, bone and brain. She takes edoxaban 30mg once daily for stroke prevention in the context of AF with a CHADSVASc score of 3 and a HASBLED score of 1. She was admitted to hospital with community acquired pneumonia five days ago but, despite optimal medical therapy, her condition continued to deteriorate. Alison is reviewed by the palliative care team who feel she is likely entering the last days to weeks of life. She is beginning to struggle with oral intake, prompting you to review her medication chart with a view to reducing tablet burden.
14. As part of your medication review, what is the most likely action you will take with regards to her edoxaban prescription?
    1. Suspend edoxaban
    2. Switch to a parental anticoagulant (e.g. a low molecular weight heparin) given the swallowing difficulties
    3. Insert an NG tube to allow enteral administration of edoxaban
    4. Continue edoxaban
    5. Other
15. Assuming all other details remain unchanged but, instead of edoxaban, Alison is taking an anticoagulant that requires blood monitoring (e.g. INR monitoring for warfarin), would you be more, equally or less likely to suspend anticoagulation?
    1. I would be **more likely** to suspend an anticoagulant requiring blood monitoring
    2. I would be **equally likely** to suspend an anticoagulant requiring blood monitoring
    3. I would be **less likely** to suspend an anticoagulant requiring blood monitoring
16. Now imagine that, instead of being in the last weeks of life, Alison was felt to be in the last *months* of life. She develops a minor upper gastrointestinal bleed from a Mallory-Weiss tear and her edoxaban is held for a week. Once she is haematologically and haemodynamically stable, what are you most likely to do regarding her edoxaban prescription?
    1. Restart her edoxaban once stable
    2. Continue to suspend edoxaban for the remainder of her life
    3. Other (please elaborate)
